# Racial Disparity in Pediatric Radiography for Forearm Fractures

**DOI:** 10.7759/cureus.22850

**Published:** 2022-03-04

**Authors:** Derek J Baughman, Taofeek Akinpelu, Abdul Waheed, Thomas Trojian

**Affiliations:** 1 Family Medicine, WellSpan Good Samaritan Hospital, Lebanon, USA; 2 Family Medicine, Wellspan Good Samaritan Hospital, Lebanon, USA; 3 Family and Community Medicine, Penn State University College of Medicine, Milton S. Hershey Medical Center, Hershey, USA; 4 Sports Medicine, WellSpan York Hospital, York, USA

**Keywords:** healthy equity, population health management, pediatric fractures, pediatrics, healthcare disparities

## Abstract

Introduction: The most common pediatric fractures involve the upper extremity. But there is limited study on racial disparity in diagnostic radiography for pediatric fractures. The literature has described the diagnostic accuracy of alternative diagnostic modalities with promising evidence of its ability to mitigate health inequity in primary care. Our objective was to understand if racial disparity exists in radiography for pediatric fractures.

Methods: In this four-year retrospective cohort study, we analyzed rates of radiographic imaging and abnormal radiograph detection in 4280 pediatric patients (ages 3-18 years) who presented with chief complaints of arm or wrist pain and trauma-related International Classification of Diseases 10th Revision (ICD-10) codes. We compared White children to all other races and stratified by emergency departments (ED) vs all other primary care ambulatory service lines.

Results: Non-White patients had lower imaging rate differences and lower odds receiving imaging in both ambulatory settings (0.65915, P = 0.0162; -5.4%, P = 0.0143) and in EDs (0.7732, P = 0.0369; -4.7%, P = 0.0368). Additionally, non-Whites in the ED had lower rates and lower odds of abnormal radiographs (-7.3%, P = 0.0084; 0.6794, P = 0.0089).

Conclusion: Non-White patients seen in emergency and ambulatory settings had lower imaging rates for traumatic arm and wrist pain compared to White patients, indicating a healthcare disparity in pediatric imaging. Higher-level studies investigating the effect of social determinants of health, more detailed patient data, and provider bias on facture care equity are needed to understand underlying reasons for observed differences.

## Introduction

Racial disparities in medicine are well-known. Non-White minorities make up a significant portion of the United States (US) population; they have decreased access to care and increased spending, especially in emergency and inpatient care [1-4]. These disparities also exist in pediatric populations. It is well described in the literature that pediatric insurance status impacts the amount of primary care received [5]. Compared with Whites, racial minorities have lower levels of health insurance coverage [6] and higher levels of cost-sharing, preventing low-income, highest-need populations from receiving needed care [7]. In addition, cost-sharing (i.e., higher out-of-pocket costs) is associated with worse treatment compliance and higher hospitalization rates [8] - a feed-forward mechanism that inflates cost burden.

Efforts to mitigate costs and increase accessibility have emerged because of the disproportionate use of emergency services by racial minorities [1]. Arm fractures are among the top reasons that pediatric populations use the emergency room/emergency department (ED) [8]. Forearm fractures in pediatric populations account for up to 50% of all fractures seen in children [9]. The known disadvantages to traditional radiography include higher cost burden, longer lengths of stay, impaired patient experience, and unnecessary ionizing radiation exposure. Accordingly, alternative diagnostic modalities to traditional radiography have gained recognition in the literature. Current evidence suggests that point-of-care ultrasound (POCUS) has comparable accuracy as radiography in diagnosing upper extremity fractures in children [9]. 

The advantages of POCUS include avoiding ionizing radiation, enhancing patient accessibility, and decreasing length of stay [9]. Moreover, POCUS can visualize indirect signs of fracture (hematoma, periosteum detachment) and evaluate soft tissue damage such as muscle edema and tendinopathy [10]. Specifically, POCUS can improve patient comfort in children, expedite diagnosis, and decrease cost without jeopardizing care [9]. In the hands of experienced providers, POCUS in primary care can detect pediatric forearm fractures, and this technology can potentially improve health equity substantially [11]. 

Although the literature has described access and cost barriers for pediatric minorities, there is limited evidence regarding racial discordance in pediatric forearm care. Additionally, little is known about the diagnostic accuracy of pediatric fractures within racial minorities - a critical step to understanding if the need exists for improved access to imaging. Thus, our study aim was to discover if there is racial disparity in the access to imaging or the detection rate of pediatric forearm fractures in an expanded health system.

## Materials and methods

We retrospectively examined the imaging and abnormal radiograph rates of upper extremity fractures in pediatric patients in the age group of 3-18 years. SlicerDicer is a self-service cohort query tool in EPIC (Epic Systems Corporation, Verona, Wisconsin) that allows users to analyze patient population-level data. A patient data model was used in SlicerDicer to mine patient visits throughout the health system from April 1, 2017, to July 31, 2021. Only the emergency services and pertinent ambulatory service lines were selected for inclusion. We measured if patients received imaging (x-ray, CT, or MRI), and if a subsequent fracture diagnosis code was added within three days of this imaging. In addition, we compared imaging rates obtained in those patients that self-identified as White and patients that did not self-identify as White, then stratified this comparison by venue (ED or Ambulatory). 

The WellSpan Health Human's Protections Office deemed this study exempt from full review (1804211-1). The Strengthening the Reporting of Observational Studies in Epidemiology (STROBE) format for cohort studies was followed.

**Figure 1:**
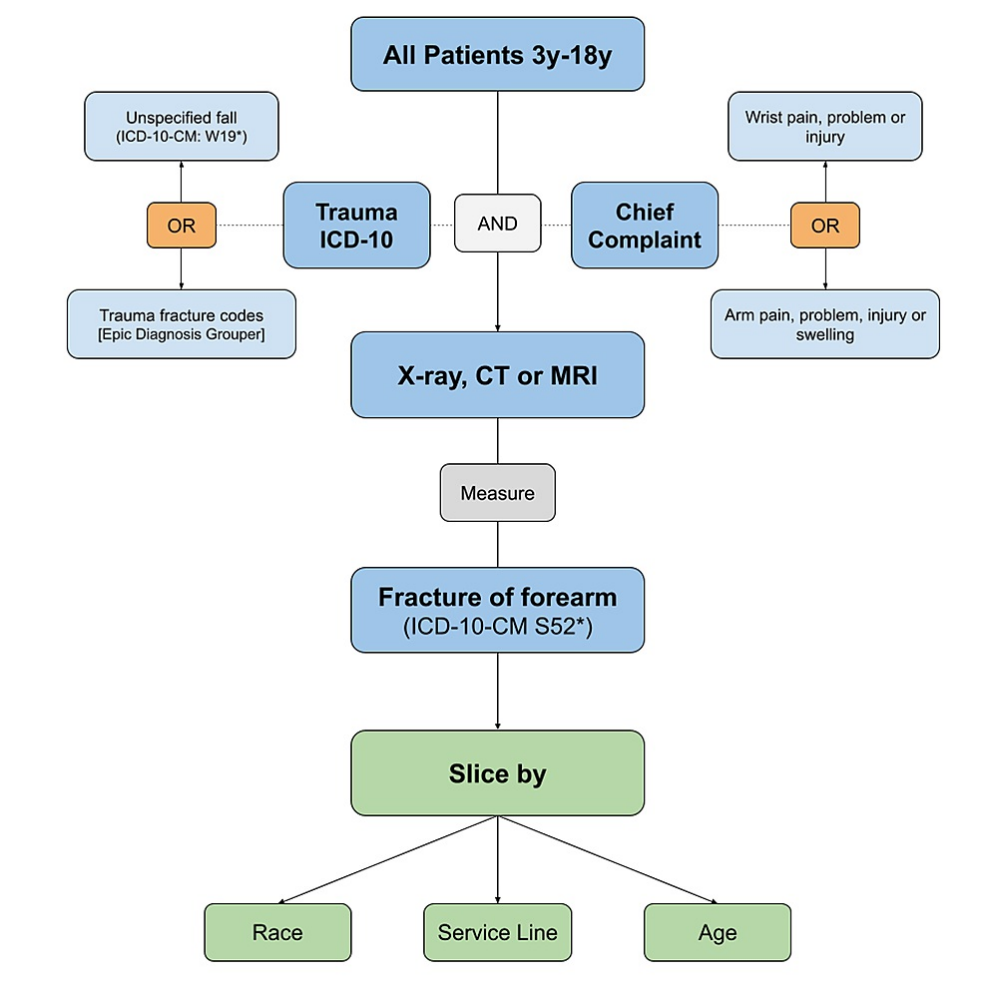
SlicerDicer patient data model schema with filter prioritization ICD-10: International Classification of Diseases 10th Revision; ICD-10CM S52*: ICD-10 Diagnosis Code S52 (Fracture of forearm); ICD-10-CM: W19*: ICD-10 Diagnosis Code W19 (Unspecified fall)

The data search session in EPIC was built according to the schema represented in Figure 1. To ensure that we accurately captured etiologies that warrant imaging for forearm fractures, ICD-10 code groupers for fall (ICD-10-CM W19*) and trauma (including V, Z, and T code categories) were added. Thus, the search comprehensively linked trauma-related codes to the chief complaint, facilitating the exclusion of non-traumatic etiologies that would have otherwise falsely inflated the denominator (for example, encounters for hand pain or superficial lacerations).

To calculate the abnormal imaging rate, in the denominator, we included all patients with chief complaints and traumatic ICD-10 codes. In the numerator, we measured all patients who had radiographic imaging completed at the same encounter and who were diagnosed with a forearm fracture (EPIC groupers for forearm fractures were used to capture ICD-10-CM S52* diagnostic codes in SlicerDicer). To avoid redundantly counting patients who may have had multiple radiographic images within the four-year timeframe, we linked fracture diagnosis measurement to a three-day timeframe. This method ensured that the image counted in the numerator was unique to the patient encounter where the image was ordered. In addition, the three-day window was designated to accommodate reflection of "read" status in the electronic medical records (EMR); this avoided underrepresenting the numerator during the brief time when imaging was completed but not officially documented by radiologists.

The conglomerate term "non-White" was used to include a representation of racial categories in the sample: Asian, Black, or African American, American Indian or Alaska Native, Native Hawaiian or Other Pacific Islander, "unknown," and "other." This was due to very limited numbers of racial categories for adequate sub-comparisons (for example, the “unknown” category contained only 13 patients).

We identified care venue as a potential bias in our study given the inherent nature of the ED and lower threshold for obtaining imaging. However, we controlled this by stratifying our data by care venue - emergency service lines vs. all other ambulatory primary care service lines.

Statistical analysis

We used gross numbers of patient encounter data obtained from SlicerDicer within Microsoft Excel (Microsoft Corp., Redmond, Washington) to calculate both rates of imaging and diagnosis and rate differences between sub-groups. To obtain subgroups, we "sliced" the results for patients according to age, race, and care venue (Figure 1). Then, Medclac's "N-1" Chi-squared calculator (MedCalc Software Ltd, Oostende, Belgium) [12] was used to determine statistical significance (P <0.05). In Chi-squared tests, we compared White patients to all other races.

## Results

Table 1 reveals the demographic data of the pediatric population. Patients presenting for traumatic arm and wrist pain were predominantly White (80.1%) with the highest representation in the age group of 14-18 years (41.5%), followed by ages 10-14 years (32.1%) and ages <10 years (26.4%). Most of the encounters were in emergency settings (79.4%).

**Table 1:** Summary of pediatric patient access and diagnostic accuracy of imaging for chief complaints of arm or wrist “pain," “injury,” or “problem” from April 1, 2017, to July 31, 2021 * Defined as (total number of patients imaged/total of patients with CC), serves as a proxy to understand the impact of care access. ± Defined as (total number of patients diagnosed with a forearm fracture/total number of patients imaged), serves as a proxy to understand impact of unnecessary (wasteful) imaging. ‡ Total pediatric patients were arrived at by tabulating race counts as this characteristic did not change over the timeframe. Note that total age group numbers sum to the matching total by race.

	Total with traumatic arm/wrist pain	Total patents imaged	Total with diagnosed fracture	Imaging rate *	Abnormal radiograph rate ±
Total patients ‡	4280	1914	504	44.7%	26.3%
White patients	3485	1539	425	44.2%	27.6%
Non-White patients	795	375	79	47.2%	21.1%
Age total	4280	1904	505	44.5%	26.5%
Age <10 years	1159	502	181	43.3%	36.1%
Age 10-14 yrars	1333	611	184	45.8%	30.1%
Age 14-18 years	1788	791	140	44.2%	17.7%
Service line totals	4280	1914	504	44.7%	26.3%
Emergency services	1972	1520	416	77.1%	27.4%
White patients	1520	1188	344	78.2%	29.0%
Non-White patients	452	332	72	73.5%	21.7%
Non-emergency services total	2308	394	88	17.1%	22.3%
White patients	1965	351	81	17.9%	23.1%
Non-White patients	343	43	7	12.5%	16.3%
Payer type totals	4280	1914	504	56.26%	26.79%
Commercial (Highmark, WellSpan, Blue Cross + other smaller payers)	2,392	1,062	298	44.38%	28.10%
Medicaid & Government	1,723	784	190	45.52%	24.25%
Self Pay/Other	165	68	16	41.39%	22.79%

In comparing service lines, there were significantly higher rates in the ED compared to ambulatory service lines for both imaging rates (ED: 77.1%, Ambulatory: 17.1, difference: 60.0 %; CI: 57.5175% to 62.3316%, Chi-squared: 1548.047, P < 0.0001) and abnormal radiograph rates (ED: 27.4%, Ambulatory: 22.3%, difference: 5.1 %; 0.2182% to 9.5674%, Chi-squared: 4.191, P = 0.0406). In combining service lines (ED + ambulatory), there were insignificant differences between Whites versus non-Whites for imaging rates (ED: 44.2%, Ambulatory: 42.7%, difference: 3.01%; CI: -0.8144% to 6.8534%, Chi-squared: 2.372, P = 0.1235) and but there were significant differences for abnormal radiograph rates (ED: 26.7%, Ambulatory: 21.1%, difference: 6.5%; CI: 1.5807% to 10.9575%, Chi-squared: 6.565, P = 0.0104).

When stratified by care venue, non-Whites consistently had lower imaging rates and lower abnormal radiograph rates (Table 2). Imaging rate differences were statistically significant in the ED (White: 78.2%, non-White: 73.5%; difference: 4.7%, P = 0.0368) and in ambulatory service lines (White: 17.9%, non-White: 12.5%; difference: 5.4%, P= 0.0143). Abnormal radiograph rate differences were statistically significant in the ED (White: 29.0%, non-White: 21.7%; difference: 7.3%, P= 0.0084) but not in the ambulatory service line (White:23.1%, non-White: 16.3%; difference: 6.8 %, P= 0.3130).

**Table 2:** Statistical analysis of access and accuracy of diagnostic imaging for forearm fractures in pediatric patients aged 3-18 years with stratification by race and healthcare venue Results stratified by treatment venue (ED vs all other ambulatory primary care venues), revealing a discordance favoring Whites over non-Whites in both imaging rate and the detection of an abnormal radiograph. * Defined as (total number of patients imaged/total of patients with chief complaint), serves as a proxy to understand the impact of care access. ± Defined as (total number of patients diagnosed with a forearm fracture/total number of patients imaged), serves as a proxy to understand impact of unnecessary imaging in pediatric patients.

		White	Non-White	Chi sq Δ	Confidence interval	P-value
Emergency Services Only	Imaging rate *	78.20%	73.50%	4.7 % Chi-squared: 4.360	(0.2854% to 9.4023%)	0.0368
	Abnormal radiograph rate ±	29.00%	21.70%	7.3 % Chi-squared: 6.946	(1.9332% to 12.1762%)	0.0084
Ambulatory Services Only	Imaging rate *	17.90%	12.50%	5.4 % Chi-squared: 6.005	(1.1534% to 8.9545%)	0.0143
	Abnormal radiograph rate ±	23.10%	16.30%	6.8 % Chi-squared: 1.018	(-7.4981% to 16.2177%)	0.313

## Discussion

We found evidence of healthcare disparity in pediatric fracture care in our health system. For traumatic forearm pain in primary care (both in emergency departments ‒ EDs and in ambulatory service lines), non-White patients had significantly lower imaging rates than White patients. In addition, non-Whites who were imaged in the ED had lower abnormal radiograph rates than Whites ‒ lower yield imaging in this care venue for non-Whites. The trend is consistent: Whites received more radiography, and that radiography was of higher diagnostic yield.

The difference in imaging rates is perhaps most meaningful in the ED sub-group since, in our study, Whites in EDs were imaged more often and with higher testing yield than non-Whites regardless of trauma. But non-Whites received 4.7% less imaging for comparable traumatic arm/wrist pain. Moreover, even if non-Whites were imaged, they had a 7.3% lower abnormal radiograph detection rate. We interpret this as evidence of healthcare disparity [2] ‒ a decrease in both the likelihood of imaging and the likelihood of diagnostic yield among non-White pediatric patients.

In ambulatory sub-group analysis, we found a similar healthcare access disparity ‒ lower imaging rates for non-Whites. However, the difference in abnormal radiograph rate was not statistically significant in the ambulatory sub-group. This difference may be less relevant in the ambulatory setting since most imaging in our study was done in EDs (there was a 4.5 fold greater rate of imaging in the ED compared to ambulatory settings). The interpretation of the ambulatory data should be in the context of care venue bias and workflows (discussed below). Thus, what is most important from the ambulatory perspective is the consistency of racial discordance in imaging rates across care venues. Non-Whites were consistently less likely to be afforded imaging despite equivocal traumatic chief complaints and diagnostic codes, regardless of treatment setting. These imaging rates could be interpreted as a proxy for care access, especially since the inclusion criteria for both racial groups were identical. These findings support the allusion to healthcare access disparity in children with suspected forearm fractures. 

Although overall, there were insignificant differences in imaging rates between races, we argue that combining emergency and ambulatory service lines may not be a valid comparison due to differences in workflows in these care venues. For example, suppose a child with traumatic arm pain enters the ED vs. a walk-in medical clinic. In that case, the inherent nature of the venue might play a meaningful role in the treatment approach for the standard of care (i.e., there might be higher rationality in ordering imaging in an emergency setting compared to an ambulatory setting). Support for this concept could be observed by comparing and approximating imaging rates between ambulatory and emergency venues from National Ambulatory Medical Care Survey data [13,14]. Workflow differences in care venues might explain the 60% higher imaging rate in the ED (Table 2).

 Locally, low yield imaging increases the likelihood of non-Whites encountering financial strain [15]. Historically, and particularly in emergency settings, the literature has supported an increased susceptibility to financial strain on marginalized populations [3,6,7,16]. Low yield imaging is medically wasteful; it provides low-value care by increasing the cost burdens like copays, deductibles, and other cost-sharing charges [17]. Although radiography may have small copays or deductibles, such a bill could be a relatively substantial percentage of weekly earnings for low-income families. Moreover, as the literature has described, most children imaged for traumatic arm wrist pain are not diagnosed with fractures, demonstrating the financial futility [9] ‒ a generalizable concept given our population's demographic similarity to US census data [18]. These types of unnecessary cost burdens imposed on vulnerable populations can augment the underlying mechanism of healthcare disparity. Thus, our study identifies a possible feed-forward mechanism that restricts care access and might contribute to disproportionate cost-sharing for non-Whites.

It is unclear why this racial disparity exists. In our study, it may be due to the disproportionately higher use of ED by non-Whites (56.9% vs. 43.6%, Table 1). It could also be that fracture rates are higher in Whites, a concept supported by the literature [19,20]. Alternatively, perhaps there is an unconscious bias that fuels the known treatment disparity in fracture care [21], or other patient and visit level factors [22]. Regardless, non-Whites received less imaging than Whites in all care venues for painful, traumatic injuries in our study. It was also unclear whether an underlying implicit bias plays a role in the decisions to image non-Whites. Future studies that could detect these concepts would be helpful for understanding structural racism in healthcare.

Identifying these disparities provides an opportunity to discuss improvement strategies for mitigating health inequity. One such strategy is increasing the use of more affordable alternative diagnostic modalities, such as POCUS, in diagnosing forearm fractures. As an established non-inferior diagnostic tool to x-ray, POCUS could be a feasible means to mitigate cost inequity [11]. Especially in rural and underserved care contexts where high racial minority representation exists [4], strategies like POCUS could effectively eliminate cost-sharing since radiologists would not be needed (and thus not billed). Moreover, since rural and underserved areas have lower access to healthcare resources [7], namely radiographic imaging services, implementation of non-radiographic options like POCUS equipment would be a substantially lower cost compared to x-ray, CT, or MRI [17]. Given the high-level evidence specifically supporting the use of POCUS in primary care for fracture diagnosis [23,24], care access for marginalized populations could be increased without the imposition of cost-sharing. 

Another strategy could be investing in alternative diagnostic modalities to promote value-based care [25]. Especially in rural practice, ultrasound has lower implementation costs with comparable sensitivity to radiography [26]. Moreover, from a patient perspective, these alternative modalities can reduce harm by decreasing radiation exposure [27,28] and improve the patient experience by reducing psychological stress [29]. The return on investment for practices and health systems could be lower costs, reduction in harm, and mitigation of financial burden for racially marginalized pediatric patients.

Limitations

First, this study presents evidence of healthcare disparity. We do not know why the imaging rates between White and non-White patients were different. Since traumatic arm/wrist pain was common to both groups, we expected imaging rates to be similar (or at least not different). Our methods lacked the capacity to detect granular data between cohorts (for example, injury mechanism, the severity of pain on presentation, time since injury, social determinants of health, etc.) which would be helpful in ascertaining what the differences between cohorts really means. Similarly, we were unable to determine if there was a correlation between the sub-categories of age and race to know if this influenced the differences in imaging or fracture detection rates; but this would require regression and additional granular data acquisition that was not accessible with our current methodology. Thus, we cannot draw conclusions on care quality and are limited to simply identifying a healthcare disparity.

Second, we could not reliably determine diagnostic accuracy. For example, there was no way of detecting whether fractures were missed or if there is a racial component affecting imaging rates (i.e. if White patients are over-imaged, have weaker bones, or have more exposure to trauma). The only reliable way to conclude diagnostic accuracy would be to review each radiograph in the study, which is beyond the scope of author practice. Thus, the authors reported abnormal radiograph rates in attempts for it to serve as a proxy for medical waste (i.e., the financial impact on marginalized populations). 

Lastly, we understand the limitations of our methods in using health system data. In relying on EMR data, there may be uncaptured or inaccurately documented visits. Also, the EMR is not equipped to assess the bias of practitioners ordering the radiography. If implicit bias played a role in racial selection, we did not have a way to measure it. Randomization would undoubtedly provide more substantial evidence to mitigate bias in future studies, which could further evaluate the appropriateness of imaging between races.

## Conclusions

This study presents initial evidence of lower imaging rates in non-White patients for traumatic arm and wrist pain compared to White patients. These differences identify a healthcare disparity in pediatric forearm fracture care, both in emergency and ambulatory settings. Implications of these findings call for higher level studies that could investigate the effect of social determinants of health, incorporate more detailed patient data, evaluate the effect of alternative diagnostic modalities, and examine provider bias on facture care equity to understand underlying reasons for observed differences.
